# The Effect of Hydrophobic Monoamines on Acid-Sensing Ion Channels ASIC1B

**Published:** 2015

**Authors:** E. I. Nagaeva, N. N. Potapieva, D. B. Tikhonov

**Affiliations:** Sechenov Institute of Evolutionary Physiology and Biochemistry, Russian Academy of Sciences, Prosp. Toreza, 44, 194223, St.Petersburg, Russia

**Keywords:** ion channels, ASIC, 9-aminoacridine, memantine, patch clamp, potentiation, inhibition

## Abstract

Acid-sensing ion channels (ASICs) are widely distributed in both the central and peripheral nervous systems of vertebrates. The pharmacology of these receptors remains poorly investigated, while the search for new ASIC modulators is very important. Recently, we found that some monoamines, which are blockers of NMDA receptors, inhibit and/or potentiate acid-sensing ion channels, depending on the subunit composition of the channels. The effect of 9-aminoacridine, IEM-1921, IEM-2117, and memantine both on native receptors and on recombinant ASIC1a, ASIC2a, and ASIC3 homomers was studied. In the present study, we have investigated the effect of these four compounds on homomeric ASIC1b channels. Experiments were performed on recombinant receptors expressed in CHO cells using the whole-cell patch clamp technique. Only two compounds, 9-aminoacridine and memantine, inhibited ASIC1b channels. IEM-1921 and IEM-2117 were inactive even at a 1000 μM concentration. In most aspects, the effect of the compounds on ASIC1b was similar to their effect on ASIC1a. The distinguishing feature of homomeric ASIC1b channels is a steep activation-dependence, indicating cooperative activation by protons. In our experiments, the curve of the concentration dependence of ASIC1b inhibition by 9-aminoacridine also had a slope (Hill coefficient) of 3.8, unlike ASIC1a homomers, for which the Hill coefficient was close to 1. This finding indicates that the inhibitory effect of 9-aminoacridine is associated with changes in the activation properties of acid-sensing ion channels.

## INTRODUCTION

A proton is the simplest neurotransmitter [1]; its effect is mediated by acid-sensing ion channels (ASICs). ASICs are voltage-insensitive channels that belong to the superfamily of degenerin/epithelial sodium channels (DEG/ENaC) and are activated in response to acidification of an extracellular medium. Currently, four genes (*accn1–4*) encoding six different ASIC subunits are known: ASIC1a and ASIC1b, which are products of alternative splicing of the *accn2 *gene; ASIC2a and ASIC2b, which are products of alternative splicing of the *accn1 *gene; as well as the ASIC3 and ASIC4 subunits [2]. A functionally active channel can be both homo- and heterotrimeric [3], with only the ASIC1a, ASIC1b, ASIC2a, and ASIC3 subunits being able to form functioning homomeric channels.

In the central nervous system, the ASIC1a, ASIC2a, and ASIC2b subunits are mainly expressed in the hippocampus, amygdala, cerebellum, striatum, cerebral cortex, and olfactory bulbs [4-10]. In the peripheral nervous system, the ASIC1b and ASIC3 subunits predominate. They can be found in the sensory neurons of the spinal cord dorsal roots and trigeminal and vagus nerves. It is worth noting that only ASIC3 can produce a sustained current in response to decrease in pH. This subtype of proton-activated channels, as well as ASIC1b, is responsible for the perception of pain stimuli accompanying an inflammation, fractures, tumors, hematomas, and postoperative wounds, and it is also involved in mechanosensation [11, 12]. In the central nervous system, ASICs are involved in important physiological processes such as synaptic transmission, synaptic plasticity, memory, learning [13], anxiety and depression [14], drug addiction [15], and chemosensation [16].

Despite the widespread occurrence of proton-activated channels in CNS and PNS, the pharmacology of these receptors remains little-studied. For example, it is known that only ASIC1a and ASIC3 homomers can be specifically inhibited by psalmotoxin-1 (PcTx1), a toxin from the venom of the South American tarantula* Psalmopoeus cambridgei *[17], and the APETx2 toxin from the venom of the sea anemone *Anthopleura elegantissima *[18], respectively. The psalmotoxin-1 specificity is lost as its concentration increases: at concentrations above 3 nM, it can also inhibit ASIC1a/2b heteromers, and at concentrations greater than 100 nM, it causes potentiation of ASIC1b [19]. The most known blocker of acid-sensing ion channels, amiloride [20], affects all types of ASICs, as well as other sodium channels of the DEG/ENaC family [21]. All attempts to synthesize more specific amiloride-based structures with one or two amidine groups have not yielded the desired results [22, 23]. Synthetic compound, 2-guanidine-4-methylquinazoline (GMQ) is able to activate selectively ASIC3 homomers via interaction with a ligand-binding domain, which differs from the proton-binding domain [24]. Thus, to date there are a few pharmacological tools differentiating subtypes of proton-activated ion channels, and the search for new, specific inhibitors/activators is the actual problem.

**Fig. 1:**
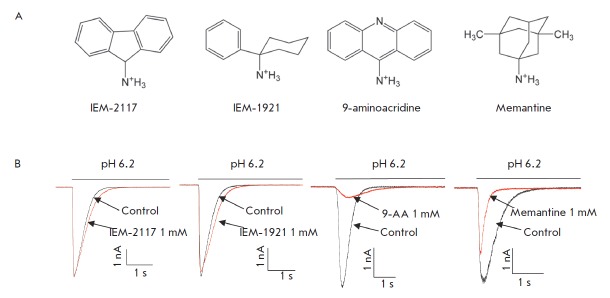
Effect of hydrophobic monoamines on ASIC1b. **A**, chemical structures of the tested compounds. **B**, representative examples of currents in the control (black) and in the presence of 1000 μM tested compounds (red)

Recently, we have demonstrated that four blockers of NMDA-receptors (*Fig. 1A*) (9-aminoacridine [25], IEM-1921 [26, 27], memantine [28], and IEM-2117 [29, 30]) can differently modulate acid-sensing ion channels, depending on their subunit composition [31]. For example, 9-aminoacridine (9AA), IEM-2117, and memantine inhibited, to varying degrees, ASIC1a homomers, while IEM-1921 had no effect even at a concentration of 1000 μM. The responses of ASIC2a, on the contrary, were potentiated by IEM-1921, IEM-2117, and memantine and were unaffected by 9AA. The effect of the tested compounds on ASIC3 was more complex because currents through these channels have peak and sustained components. IEM-1921 and 9-aminoacridine potentiated the sustained component but inhibited the peak component. IEM-2117 and memantine potentiated both components of the response. In this case, IEM-2117 was the most active potentiator and it also activated ASIC3 channels in a neutral pH (7.4), causing a sustained current.

In the present work, we studied the effect of four compounds mentioned above on a homomeric channel formed by the ASIC1b subunit, which is a product of alternative splicing of the *accn2 *gene. This channel is interesting because of its very specific activation curve with a high Hill coefficient (*n*_H_) equal to 4.8 [32]. Analysis of the effects of potentiators/inhibitors on this receptor may help test the hypothesis of a possible mechanism of ligand action via increasing/reducing affinity of protons for the proton binding site of ASICs. We have demonstrated that the effect of hydrophobic monoamines on ASIC1b is similar to their effect on ASIC1a, except that the concentration-dependent inhibition curve of 9AA has a much greater Hill coefficient compared to that for ASIC1a.

## EXPERIMENTAL

CHO cells (Chinese hamster ovarian epithelial cell culture) were cultured in a CO_2_ incubator at 37 °C and 5% CO_2_. The cell growth medium consisted of a DMEM/F12 (Dulbecco’s Modified Eagle’s Medium) solution supplemented with 10% fetal bovine serum and 1% streptomycin/penicillin. Transfection of cells with plasmids was performed using the Lipofectamine 2000 reagent (Invitrogen, USA) according to the manufacturer’s protocol. The plasmid carrying the rASIC1bpECFP- C1 construct was courtesy of A. Starushchenko. CHO cells were seeded on glasses with an area not exceeding 25 mm^2^ and uniformly distributed on the bottom of a Petri dish with a diameter of 35 mm. For the expression of homomeric ASIC1b channels, the cells were transfected with the plasmid (0.5 μg) carrying the *ASIC1b *gene, together with the plasmid (0.5 μg) encoding the fluorescent protein GFP. Electrophysiological experiments were performed 36–2 h after transfection. Transfected cells were detected by green luminescence using a Leica DM IL microscope (Leica Microsystems, Germany).

The currents caused by fast acidification of the medium were recorded using the whole cell patch clamp technique. For this purpose, an EPC-8 amplifier (HEKA Electronics, Germany) was used; the signal was filtered in the frequency band of 0–5 kHz, digitized at the sampling rate of 1 kHz and recorded on a personal computer using the Patchmaster software from the same manufacturer (HEKA Electronics, Germany). All experiments were performed at room temperature (23–25 °C). The micropipette solution contained 100 mM CsF, 40 mM CsCl, 5 mM NaCl, 0.5 mM CaCl_2_, 5 mM EGTA, and 10 mM HEPES (pH was adjusted to 7.2 by adding CsOH). The extracellular solution contained 143 mM NaCl, 5 mM KCl, 2.5 mM CaCl_2_, 10 mM* D*-glucose, 10 mM HEPES, and 10 mM MES (pH was adjusted to 7.35 by adding NaOH). All solutions were filtered through micropore cellulose membranes using a vacuum glass filter (Sartorius AG, Germany).

Solutions with low pH values, which were used to activate channels, were prepared from the extracellular stock solution by adding HCl. The monoamines were synthesized earlier, under a reques from our laboratory, by V.E. Gmiro at the St. Petersburg Institute of Experimental Medicine. To prepare a stock solution with a monoamine concentration of 5 × 10^-2^ M, a sample weight of its crystalline form was dissolved in bidistilled water. Further, the required volume of the stock solution was added to working solutions with different pH values. When preparing monoamine solutions, the pH of the resulting mixture was checked for each preparation. If a shift was detected, then the pH was adjusted to the required value using a 0.1 N HCl solution or a 0.2 N NaOH solution. For fast drug application the ALA-VM8 manifold system (ALA Scientific Instruments, USA) was used. The interval between test applications was 60 s.

All data are presented as a “mean ± standard deviation” calculated from at least five experiments. The statistical significance of the effects was evaluated using the paired *t*-test with *p *= 0.05 (the value of the response amplitude in the presence of a test compound relative to the control). The response shape was analyzed by measuring the current rise time from 10 to 90% of the maximum amplitude and calculating the response decay time constant using a least squares exponential fitting.

**Fig. 2:**
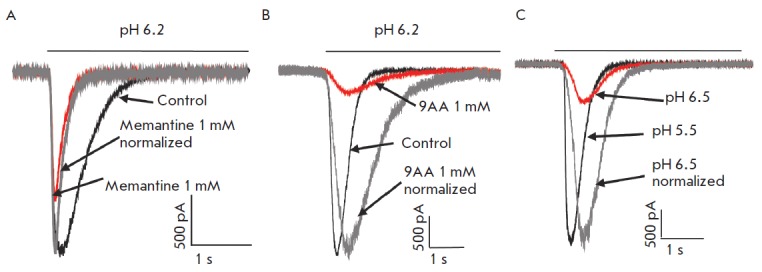
Changes in the response kinetics in the presence of 1000 μM memantine (**A**) and 1000 μM 9AA (**B**). The gray trace shows the response in the presence of an inhibitor. The response is normalized by the amplitude to the control level. Memantine, a weak inhibitor, increases the rate of desensitization. Contrary, 9AA broadens the response. **C**, examples of the currents evoked by modest (red) and strong (black) acidifications. As in the presence of 9AA (**B**), response to modest acidification has a low amplitude and slow kinetics

To simplify evaluation of the changes in the response kinetics under the influence of the test compounds, the currents were normalized by the amplitude (*Fig. 2*). For this, the ratio of the control current in response to pH and the current in the presence of a test compound was calculated. The current with the smaller amplitude was multiplied by the obtained ratio, thereby producing responses with equal amplitudes.

## RESULTS

**Fig. 3:**
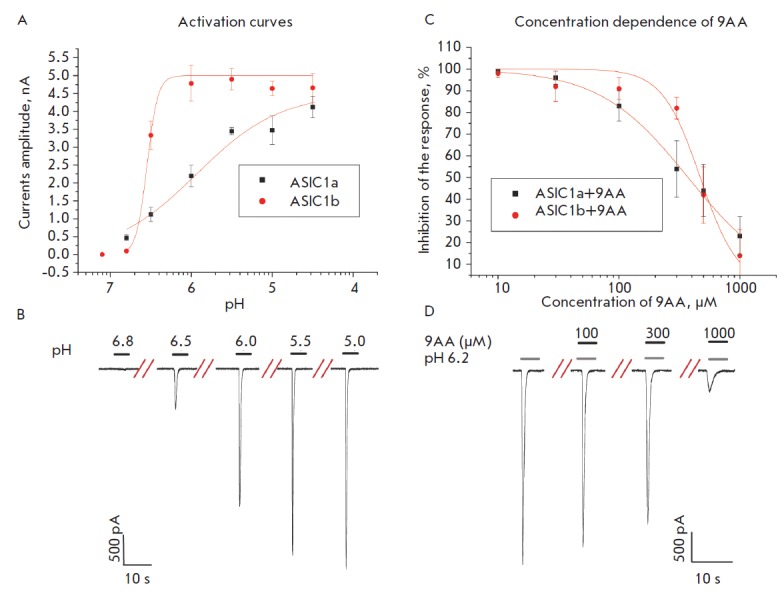
Correlation between the activation properties of ASIC1a and ASIC1b and their inhibition by 9AA. **A**, pH-dependencies of the response amplitude for ASIC1a (black dots) and ASIC1b (red dots) activation. **B**, representative examples of ASIC1b channel currents evoked by different pHs. The interval between applications is 60 s. **C**, concentration dependencies of ASIC1a (black dots) and ASIC1b (red dots) inhibition by 9AA. **D**, representative examples of ASIC1b currents at pH=6.2 in the presence of different 9AA concentrations

Reducing the pH of the extracellular medium from the initial level of 7.35 resulted in transient currents in cells carrying the ASIC1b plasmid. Threshold currents exceeding the noise level by more than 2 times (40–100 pA) were observed for a solution with pH= 6.8. When the solution pH was reduced to 6.5 (*Fig. 3A,B*), currents up to 1 nA were detected. This sharp increase in the response is related to the high slope of the activation curve (*n*_H_ = 4.9 ± 0.2; pH_50_ 6.3 ± 0.2, *n *= 5) (*Fig. 3A*). These results are consistent with previously published data [32]. The classical blocker of acid-sensing ion channels, amiloride, (30 μM) blocked 53 ± 7% (*n *= 6) of the currents evoked by application of a solution with pH=6.2. The kinetics of the response decay due to receptor desensitization (τ = 0.67 ± 0.12 s, *n *= 5) was also consistent with the previously published data.

None of the four tested compounds caused currents in the neutral medium even at high concentrations (data are not shown).

**IEM-1921 and IEM-2117** Since ASIC1b and ASIC1a are two alternative splice variants of the same *accn2 *gene, it can be assumed that the effect of the compounds on ASC1b will be similar to those on ASIC1a. However, this assumption was correct only for some of the compounds. As in the case of ASIC1a, a phenylcyclohexyl derivative IEM-1921 exhibited no activity on ASIC1b channels at concentrations ranging from 10 to 1000 μM. The effect of IEM-2117 was the same (*Fig. 1B*), although, in the case of ASIC1a, it acted as a weak inhibitor: 1000 μM of the compound caused 34 ± 10% (*n *= 7) decrease of the response.

**Memantine**

The only clinically used blocker of NMDA receptors [33], memantine, had no effect on ASIC1b homomers at concentrations below 100 μM. However, at higher concentrations, memantine behaved as a weak inhibitor. Thus, memantine at a concentration of 300 μM inhibited 19 ± 6% (*n *= 5) of the current, and application of 1000 μM resulted in 44 ± 16% (*n *= 5) decrease in the response amplitude (*Fig. 1B*). Since a saturating concentration of the compound was not achieved, it was impossible to measure the IC_50_ parameter. Apart from the inhibitory effect, 1000 μM memantine induced a decrease in the response decay time constant from 0.50 ± 0.12 s (*n *= 6) to 0.15 ± 0.02 s (*n *= 5) (*Fig. 2A*). Earlier, we had observed a similar change in the response shape for ASIC1a homomers.

**9-Aminoacridine**

9AA was the most potent inhibitor of ASIC1b channels. 1000 μM 9AA reduced the response amplitude by 86 ± 10% (*n *= 7) upon simultaneous application with a solution with pH 6.2 (*Fig. 1B*). IC_50_ was 440 ± 20 μM (*n *= 7) (*Fig. 3C*). An interesting feature of the 9AA effect on ASIC1b channels was a sharp increase in the inhibitory effect upon a slight increase in the compound concentration; i.e., the Hill coefficient was high (3.8 ± 0.5, *n *= 5) (*Fig. 3C*). The curve of ASIC1b sensitivity to the agonist is also characterized by a high Hill coefficient (see above). On the contrary the curves of ASIC1a activation and its inhibition by 9-aminoacridine had a Hill coefficient of 1.2 ± 0.3 (*n *= 5) and 1.3 ± 0.3 (*n *= 5), respectively.

9AA significantly changed the shape of the ASIC1b response to acidification (*Fig. 2B*). In the presence of 1000 μM 9AA, the response kinetics became slower and the current rise time increased from 0.15 ± 0.02 s (*n *= 5) in the control to 0.48 ± 0.12 s (*n *= 5). The response decay time constant also increased significantly (τ = 0.67 ± 0.12 s, *n *= 5 in the control and τ = 1.2 ± 0.2 s, *n *= 5 in the presence of 9AA). This effect may be caused by asynchronous activation of channels, which is typical of the action of low agonist concentrations. Indeed, a similar difference was observed upon ASIC1b activation by acidification to pH 6.5 and 5.5 (*Fig. 2C*); i.e., it may be proposed that channel affinity for protons decreases in the presence of 9AA. Therefore, in the presence of 9AA, the amplitude and shape of the response to the solution with pH 6.2 become similar to those of the response to the solution with pH 6.5.

**Fig. 4:**
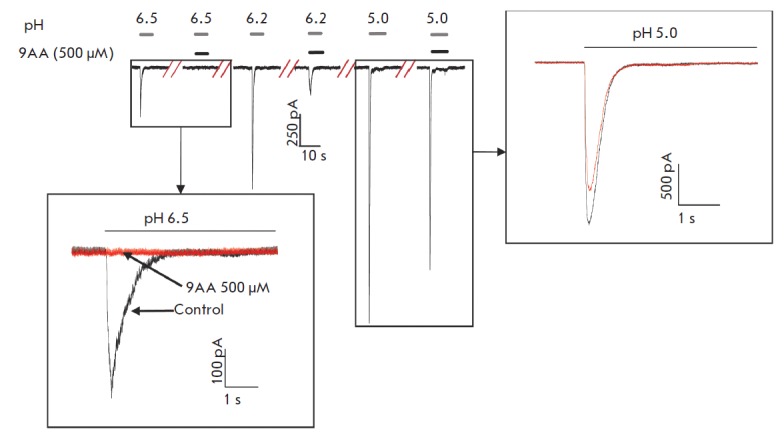
Dependence of the 9AA effect on the ASIC1b activation level. Representative examples of currents through ASIC1b are shown for different pH values in the presence and absence of 500 μM 9AA. Insets show superimposed currents at a larger scale. At pH=6.5 (lower inset), inhibition is almost complete. At more acidic pH, which causes strong ASIC activation (right inset), inhibition becomes modest

Since the effect of 9-aminoacridine on ASIC1a homomers was previously characterized by a pronounced pH-dependence (weakening of inhibition as the activating pH value decreased), we decided to analyze this effect on ASIC1b channels, too. Under conditions of a relatively low proton concentration (pH 6.5), an almost complete response inhibition (92 ± 3%, *n *= 7) was observed. Upon stronger acidification (pH 5.0), the effect decreased to 28 ± 8% (*n *= 5) (*Fig. 4*). This fact agrees with the hypothesis of reduction of proton affinity for the receptor as a possible mechanism of 9AA action.

## DISCUSSION

As it might be expected, the effect of hydrophobic monoamines on ASIC1b homomers largely resembles their effect on ASIC1a homomers. The phenylcyclohexyl derivative IEM-1921 had no effect on the activity of both channels. Memantine and 9AA exerted a pronounced inhibitory effect upon simultaneous application with an acidic solution. Similar to the case of ASIC1a, memantine not only reduced the response amplitude, but also greatly decreased the current decay time constant. 9AA was found to be the most potent inhibitor: at a concentration of 1000 μM, it caused 86 ± 10% (n = 7) of the response via ASIC1b and 77 ± 9% (*n *= 6) of the response via ASIC1a. The effect of 9AA was characterized by a pronounced pH-dependence in both cases: the inhibitory effect considerably decreased at the saturating agonist concentration. Only IEM-2117 exhibited some subunit specificity and did not inhibit ASIC1b homomers. Despite the small differences in the effect of the tested compounds on the two related homomers, it may be concluded that alternative splicing has no direct effect on the action of hydrophobic monoamines.

At this stage, it is impossible to draw definitive conclusions about the action mechanism of the studied compounds on ASIC channels. Probably, there are differences in the action mechanisms between memantine and 9AA, since these compounds differently change the shape of the response to acidification (*Fig. 2 A,B*). The effect of memantine (decrease in the decay time constant) resembles the effect of open channel blockers or desensitization promoters. The effect of 9AA is probably associated with a change in the affinity for protons. The arguments in favor of this hypothesis are (1) the correlation between the Hill coefficients for activation of channels and their inhibition by 9AA (*Fig. 3 A,C*) and (2) the analogy between the change in the response shape in the presence of 9AA and upon channel activation by slight acidification (*Fig. 2 B,C*). More exact conclusions about the mechanisms and sites of the binding of hydrophobic monoamines to ASICs require further research.

## CONCLUSIONS

In this paper, in addition to earlier results, we have demonstrated that classical blockers of NMDA receptors can modulate the activity of all functionally active ASIC homomers and that the specificity of the effect depends on the subunit composition of a receptor. Importantly, all the tested compounds have very simple chemical structure comprising one amino group and a hydrophobic “core.” This structure differs from the amidine-containing derivatives of amiloride and other known modulators of acid-sensing ion channels. This fact makes it possible to assign hydrophobic monoamines to a new class of ASIC ligands. Furthermore, it suggests that ASICs can serve as targets for many clinically used drugs (e.g., tricyclic antidepressants and some psychotropic compounds), as well as endogenous monoamines and their derivatives. The latter suggestion is crucial in understanding the physiological role of proton-activated ion channels in the CNS. As mentioned above, ASICs have a high expression level in all of the most vital parts of the brain. However, the range of pH where these channels are activated is atypical of normal physiological processes. Therefore, there is a high probability of existence of endogenous activators/ modulators of these channels. The search for those endogenous amines seems promising.

## Abbreviations

ASIC: acid-sensing ion channel
CNS: central nervous system
PNS: peripheral nervous system
GFP: green fluorescent protein
IC50: half maximal inhibitory concentration.
